# Artificial Intelligence in Obesity Prevention

**DOI:** 10.3390/healthcare13243262

**Published:** 2025-12-12

**Authors:** Golbarg Shabani Jafarabadi, Luca Busetto

**Affiliations:** 1 Department of Agronomy Food Natural Resources Animals and Environment, University of Padova, 35128 Padova, Italy; 2 Department of Medicine, University of Padova, 35128 Padova, Italy; luca.busetto@unipd.it

**Keywords:** obesity, artificial intelligence, obesity prediction

## Abstract

**Background/Objectives:** Obesity is a complex disorder that causes further health issues linked to several chronic diseases, such as cancer, diabetes, metabolic syndrome, and cardiovascular diseases; thus, it is critical to identify and diagnose obesity as soon as possible. Traditional methods, such as anthropometric measures, were popular, although recent advances in artificial intelligence (AI) offer new opportunities for prediction models; as a result, AI has become an essential tool in obesity research. This study provides a comprehensive analysis of the research on the impact of AI on obesity prevention. **Methods**: In this study, the researchers performed a scoping study using AI to assess and predict obesity in PubMed, Scopus, Web of Science, and Google Scholar from February 2009 to July 2025. The researchers compiled and arranged the employed AI approaches to find connections, patterns, and trends that could guide further research and the application of machine learning algorithms for advanced data analytics. **Results:** Clinical professionals in obesity medicine may find chatbots valuable as a source of clinical and scientific knowledge, and for creating standard operating procedures, policies, and procedures. According to the findings, AI models can be used to identify clinically significant patterns of obesity or the connections between specific factors and weight outcomes. Moreover, the application of deep learning and machine learning approaches, such as logistic regression, decision trees, and artificial neural networks, appears to have yielded new insight into data, particularly in terms of obesity prediction. **Conclusions:** This work aims to contribute to a better understanding of obesity detection. While more studies are needed, AI offers solutions to modern challenges in obesity prediction.

## 1. Introduction

### 1.1. Obesity

Obesity should be considered a chronic progressive disease resulting from abnormal and/or excessive fat accumulation [1,2,3,4]. Cardiovascular disease (CVD) is the primary cause of death linked to obesity. Type 2 diabetes (T2D) [5], chronic renal disease, metabolic dysfunction associated with steatotic liver disease [6], hypercholesterolemia, and many kinds of cancer are the most prevalent consequences of obesity [7,8].

The prevalence of obesity has nearly tripled since 1975 and is projected to increase to 1.02 billion (18% of adults) by 2030. Nearly two billion adults, children, and adolescents, in other words, 24% of the world’s population, are projected to be living with obesity by 2035 [9].

There are many reasons leading to obesity, such as overconsumption of highly palatable, energy-dense foods and increased sedentary behavior [10]. Moreover, commercial determinants of health, including increased food availability, marketing, pricing, portion sizes, energy density, ultra-processing of foods, and prioritizing profitable by-products, as well as other societal and environmental changes, such as insufficient sleep, elevated stress, and exposure to endocrine-disrupting chemicals, are some of the hypothesized causes of increased food consumption [11]. Therefore, the fundamental reasons for the rise in obesity are multifaceted and include interactions between environmental, behavioral, social, genetic, and economic factors as well as human biology [12,13].

Many organ and tissue functions are disrupted by excess adipose accumulation, such as venous stasis; increased oxygen consumption; restricted diaphragmatic movement; pharyngeal collapsibility, which results in respiratory insufficiency and obstructive sleep apnea; increased abdominal pressure that causes gastroesophageal reflux disease; and increased wear and damage to weight-bearing joints that cause osteoarthritis, pain, and impaired mobility [14]. Depression and obesity frequently coexist and are correlated, as obesity increases the risk of depression and depression is a risk factor for obesity. People with obesity frequently have a lower quality of life; those with a higher BMI report lower physical and mental quality of life [15].

Dysregulated adipose tissue triggers a chain reaction of pro-inflammatory reactions, resulting in systemic insulin resistance, glucose dysregulation, and fatty acid dysregulation. Organs like the arteries, heart, liver, skeletal muscle, and pancreas are damaged by this dysregulation, contributing to systemic hormonal, metabolic, and target organ changes. These negative consequences of obesity are correlated with the amount and distribution of excess body weight [16].

The diagnosis of obesity is still based solely on the body mass index (BMI), although it does not show adipose tissue distribution and function in determining the severity of the disease; therefore, anthropometric assessments beyond the BMI are required for an accurate diagnosis of obesity, particularly for individuals in the intermediate BMI ranges [17,18]. Furthermore, the concept of obesity in the new paradigm includes those with a lower BMI (≥25–30 kg/m^2^) who accumulate more abdominal fat and have any functional, psychological, or medical difficulties. Due to its superior risk indicator status for cardiometabolic diseases, the waist-to-height ratio was chosen for the diagnostic procedure rather than waist circumference [19,20].

Methods for assessing body composition, including dual-energy X-ray absorptiometry (DEXA), which is considered the gold standard; hydro-densitometry; magnetic resonance imaging; and computerized tomography, are more reliable indicators of body fat percentage but are limited in use since they are expensive, labor-intensive, and may cause discomfort or danger to individuals [21].

Lifestyle modification has been established as a first-line treatment of obesity. A comprehensive lifestyle program that includes nutritional therapy, stress reduction, improved sleep, psychological therapy, obesity medications, and metabolic or bariatric (surgical and endoscopic) procedures should be performed [22]. It is recommended to encourage adherence to a healthy diet and regular physical activity for at least six to twelve months and to engage in at least 150 min of moderate-intensity exercise each week. It is recommended to make these lifestyle changes to lose weight and maintain it. Exercise and calorie restriction should, therefore, be taken into consideration [23,24,25].

Besides traditional assessment methods for obesity prediction, various artificial intelligence categories, including machine learning and deep learning, provide sophisticated tools to enable a practical approach in managing and preventing obesity.

### 1.2. Artificial Intelligence

Machine learning is designed to operate to varying degrees without direct human supervision and provides tasks with adaptive outputs based on principles from data input. Machine learning techniques can automatically identify complex, nonlinear relationships between the predictor variables and the response variable or variables. Consequently, these models’ predictive ability, usability, and resilience for complex data greatly exceed those of the conventional statistical models. It has placed more emphasis on prediction than testing a predefined hypothesis [26].

Machine Learning (ML): Machine learning is a powerful new class of highly optimized algorithms that can collectively learn, adapt, forecast, collect, and analyze data to predict various conditions, including obesity. ML can be divided into supervised and unsupervised [27].

Unsupervised Machine Learning: This examines and groups unlabeled datasets to find hidden patterns or clusters without human assistance. It has a high capacity to highlight informational similarities and discrepancies, so it is perfect for exploratory data analysis. Three primary tasks are performed by unsupervised machine learning models: dimensionality reduction, association, and clustering. Machine learning aims to find the hidden structure in the data and group it into clusters or groups [28,29]. Here are some examples of unsupervised and supervised machine learning.

K-means Clustering: The iterative K-means clustering algorithm divides the dataset into a maximum of k distinct, non-overlapping groups or clusters. Every data point belongs to a single group. While maintaining the distance between the clusters, the method seeks to maximize the similarity between intra-cluster data points [30].

Fuzzy C-means Clustering: In non-fuzzy clustering, also known as hard clustering, which includes K-means clustering, data are separated into discrete clusters to which a single data point can belong. Data points in fuzzy clustering may belong to more than one cluster [31,32].

Group Factor Analysis: Factor analysis explains the connections between a dataset’s distinct variables. This traditional approach is extended by group factor analysis (GFA), which describes correlations among variables, where each group represents a set of related variables or data [33,34].

Supervised: A training set of input-output pairs is used in machine learning so that the algorithm can learn a function that connects the input to the output [35].

Linear Regression: Although it is regarded as a traditional statistical model and a classical architecture for creating a predictive model, linear regression satisfies all ML requirements. It is a popular ML technique for predicting continuous outcomes like BMI. Its benefits are readability and simplicity [36].

Logistic regression: In its most basic form, logistic regression models a binary outcome using a logistic function known as the sigmoid function. A sigmoid function is the ideal mathematical tool for describing probabilities as it is a continuous, smooth, and differentiable S-shaped function that maps a real number to a value between 0 and 1 [37,38].

Naive Bayes (NB) Classifier: Based on the value of the class, NB algorithms apply the Bayes theorem under the naive assumption that each pair of characteristics has conditional independence. Notwithstanding this oversimplified presumption, NB classifiers are extensively employed and effectively resolve real-world issues [39].

K-Nearest Neighbors: Suitable for regression and classification applications, K-Nearest Neighbor (KNN) is a non-parametric supervised learning technique. The input comprises the k training data points closest to each other based on a predetermined distance measure. It groups data points into k clusters, minimizing intra-cluster distances and optimizing inter-cluster distances. KNN is a memory-based learning algorithm that can become noticeably slower as the sample grows. It is referred to as a lazy learner because it requires no training [40].

Support Vector Machines (SVMs): Support vector machines (SVMs) are supervised learning models that address regression and classification problems by constructing a hyperplane in a high-dimensional space [41].

Decision Trees (DTs): Decision trees are non-parametric supervised learning techniques used for regression and classification tasks. A tree is constructed using DT algorithms by dividing the source set, which forms the tree’s root node, into subsets that comprise the succeeding children [42].

Random Forest (RF) Models: Ensemble methods combine the predictions of a set of models to improve performance in tasks involving regression or classification [43].

Artificial Neural Networks (ANNs): ANNs are machine learning techniques that mimic the structure and operation of the nervous system. They are made up of interconnected layers of artificial neurons. The input variables in the first layer correlate with these variables, which are used as inputs of the neurons in the next layer. Each of these neurons produces an output that is utilized as an input to the neurons in the next layer, and so on. An output layer, composed of neurons coupled to every other neuron in the layers, and “hidden layers” make up this network [44].

Convolutional neural networks (CNNs): CNNs are the most widely used architecture in medical image processing, and the most popular models are currently trained end-to-end in a supervised manner. They include many layer types, each serving a distinct function, including convolution, pooling, and fully connected layers. CNNs focus on object detection and image classification [45].

Deep Learning (DL): This is an area within machine learning (ML) that makes use of numerous (deep) layers of artificial neurons to extract higher-level features from data gradually. Comparing this layer representation to classical machine learning (ML), which is used in analyzing large-scale, messy data, allows for modeling more intricate and dynamic patterns [46].

This review explores how AI affects obesity prediction, reshaping the field of nutrition and emphasizing how machine learning and deep learning algorithms can enhance early detection, risk assessment, and personalized intervention strategies. It may clarify how academics and clinicians can use AI-based predictive tools to develop more precise, effective, and customized approaches to managing and preventing obesity.

## 2. Materials and Methods

### 2.1. Search Strategy

This section provides a comprehensive overview of the study’s dataset, artificial intelligence, and performance standards for evaluating the models’ ability to predict obesity. We conducted this narrative review of literature focusing on artificial intelligence (AI) applications for obesity prediction. From February 2009 to July 2025, a systematic search was conducted using both Mesh terms and non-Mesh terms across online databases, including ScienceDirect, Web of Science, PubMed, and Scopus. The search strategy consisted of “Obesity” OR “Overweight” AND “Artificial Intelligence” OR “AI” OR “Machine Learning” OR “Deep Learning” OR “Machine learning techniques” OR “predictive models” AND “Obesity Prediction”. We also examined a manual search of references from the selected articles.

### 2.2. Study Selection

The selection process mainly aims to identify relevant articles. A total of nearly 894 articles were initially obtained from automatic searches based on the keywords defined above. Studies included in this review were narrowed to approximately 41 articles after eliminating repeated articles, based on inclusion criteria, and using the reference Endnote.

### 2.3. Inclusion and Exclusion Criteria

Inclusion criteria were as follows: (1) any English-written publication after 2009 that investigated the association and interaction of AI and obesity prediction, (2) original investigations applying AI or machine learning methods to predict obesity or obesity-related outcomes, (3) reported prediction accuracy, model performance, or validation, and (4) full-text articles access.

Exclusion criteria were as follows: (1) articles that addressed general AI in disease prediction without mention of obesity, (2) investigations with poor methodological quality, (3) articles that show the effect of AI on nutrition without focusing on obesity, and (4) animal studies.

## 3. Results

The way nutrition is currently provided is changing significantly due to artificial intelligence (AI). Conventional methods are being replaced by more advanced software that evaluates body weight, food intake, and diet-related diseases. Additionally, advanced data storage systems are being used to meet current demands through chatbots and mobile applications [47]. Artificial intelligence (AI) technology has been widely used to implement digital healthcare through a variety of methods, including the implementation of smart homes, the creation of multimodality interfaces for real-world applications, and the use of biomedical sources to provide users with feedback [48]. This study provides a new artificial intelligence (AI) system that simulates the way dietitians think by estimating portion sizes using a collection of everyday objects as gauges, such as a teaspoon, a golf ball, a cup, and so forth. Using the calculated probability vector and the stored volumes, this AI system generates the best-guess volume. According to these experimental findings, the human-mimetic method is reliable and accurate, generating an acceptable estimate from a 2D image that only partially contains 3D information [49].

### 3.1. Logistic Regression and Artificial Neural Networks (ANNs) in Obesity Prediction

Ferdowsy et al. employed several machine learning algorithms to predict the likelihood of obesity and highlighted the adverse effects of unhealthy lifestyles on teenagers. The study highlighted the variance in algorithm performance across many studies and found that logistic regression was the best-performing algorithm [50], although regression modeling may not be able to effectively untangle interdependent and nonlinear interactions between characteristics to predict obesity, as the causes of obesity are complex [51].

A later paper by Rios-Julián et al. attempted to predict obesity, overweight, and normal weight by using the BMI and other anthropometric variables. They modeled a cohort of 221 children, 6–13 years old, using different ML models: J48, logistic model trees, ANN, RF, and logistic regression. Three groups of variables were tested on all variables except skinfold thickness, sex, age, height, weight, and BMI. Overall, all of the models produced very good predictions, and they found that the findings for the various variable groups and models did not differ much. A 10-fold cross-validation process was used to internally validate each model [52]. Additionally, according to Thamrin et al., machine learning methods outperform logistic regression in predicting outcomes in obesity research, with an accuracy of 0.72 reported in other studies [53,54,55].

Zhang et al. investigated the comparison between ML models and the conventional logistic regression model based on historical data, and they created many models to predict overweight at three years. All child characteristics, such as gender, BMI at eight months, adjusted SDS of height at various visits, and weight growth between pairs of visits, were included in these variables. The machine learning techniques employed were DT, Association Rules, ANN, Linear SVM, radial basis function (RBF), SVM, BN, and NB. The RBF and SVM (perhaps more beneficial for clinical applications) had the most significant sensitivity in the prediction at eight months, whereas the ANN showed the most considerable accuracy. Regarding the two-year prediction, Bayesian techniques yielded the highest accuracy, while RBF and SVM showed the highest sensitivity. Logistic regression had the highest specificity compared to machine learning models, but its sensitivity and accuracy were significantly lower [56].

Lazarou et al. conducted a study in which they employed food characteristics to predict the likelihood of overweight obesity in comparison to average weight. A cross-sectional setting and a cohort of approximately 600 10- to 12-year-old Cypriot children were employed. As predictor variables, they employed questionnaires about the frequency of consumption of several food types (fried food, shellfish, delicatessen meat, soft drinks, sweets, and junk food). They were able to determine the principles governing the danger of overweight obesity as a function of sex and dietary habits by creating several DTs for both boys and girls. Bootstrapping was used to validate the method; however, the results were not displayed. Lastly, they created logistic regression models with PCA components of the diet variables as predictor variables; only one of the PCs of the girl’s model was significant [57].

In a 2014 study, Pochini et al. used logistic regression and DT in a cross-sectional scenario to predict overweight and obesity in high school adolescents (aged 14–18) based on nine lifestyle predictor variables. A cohort of about 15,000 high school students in Columbia, USA, served as the model sample. The variables that remained in the DT after pruning were tobacco and physical activity. For obesity, the logistic regression model identified significant predictors such as smoking, eating a regular breakfast, drinking fruit juice, and consuming soda. Regular breakfast consumption and physical activity were the most significant predictors of overweight. After pruning, no variable was left for the DT; the variables prior to pruning were sleep, fruit juice, and breakfast. 30% of the original sample was used for external validation of the models in the DT example [58].

Ergün et al. applied logistic regression and neural network methods for classifying obesity disease. This study used two distinct mathematical models—a multilayer perceptron (MLP) neural network and a traditional statistical method based on logistic regression—to classify the areas affected by obesity. The classification performances of the two models were then compared. This comparison reveals that neural networks outperform logistic regression in classification, and the reasons behind this difference were also examined. Additionally, it is noted that obesity has a more significant impact on body mass index, as classified in [59]. However, in another study comparing artificial neural networks with logistic regression for the detection of obesity, every participant completed questions regarding their socioeconomic standing, and a qualified nurse measured their anthropometric measurements. Artificial neural networks and logistic regression were utilized to categorize obese cases. The logistic regression and neural network models’ corresponding values were 80.2% and 81.2% for correctly classified cases, 80.2% and 79.7% for sensitivity, and 81.9% and 83.7% for specificity. Additionally, the corresponding values for the area under the Receiver Operating Characteristic (ROC) curve were 0.888 and 0.884, and the corresponding Kappa statistics were 0.600 and 0.629. They conclude that whereas logistic regression and neural networks were both effective classifiers for detecting obesity, there was no discernible difference between them in classification [60].

Duran et al.’s paper outlines the use of ANN models to forecast body fat percentage (BF%) and its excess (BF% over the 85th percentile) as an alternative to BMI-based indicators of obesity. This study used a cohort of approximately 2000 white, non-Hispanic children under the age of 20. Separate models were developed for girls and boys, with waist circumference, weight, age, and height considered as predictors. Z-BMI and Z-WC were used to compare the predictions made by the ANN. In conclusion, regarding males, artificial neural networks (ANNs) outperform simple models—particularly the Z-WC model—in terms of accuracy, sensibility, and specificity, and regarding girls, ANNs outperform both the Z-BMI and Z-WC models [61].

Ríos-Julián et al. showed that research comparing two or more machine learning algorithms revealed that the ID3 decision tree algorithm, Random Tree, and Random Forests performed similarly well in terms of accuracy, outperforming J48 (C4.5) decision trees, Naïve Bayes, and Bayes Net. This study focused on predicting obesity in children up to the age of ten. An additional study aimed at predicting obesity in teenagers discovered that weighted ANNs and K-Nearest Neighbor (KNN) performed more accurately than binary logistic regression and Improved Decision Trees (IDTs). ANNs, logistic model trees, and basic logistic regression performed better in accuracy than J48 (C4.5) decision trees and Random Forests in research for overweight–obesity screening in children up to 13 [52].

### 3.2. Naive Bayes (NB), Random Forest (RF) in Obesity Prediction

Regarding the 2010 review, Adnan published three publications in this field in 2012 using NB and a cohort of 140 Malaysian children aged 9 to 11 to predict their nutritional status (normal weight, overweight, and obese). They used 19 predictor factors from various domains—children’s characteristics, lifestyle (including food and exercise), and family/environment—that they had gathered from the literature review [62,63].

To construct the obesity dataset, 1610 people participated in an online survey. Following pre-processing, four widely used artificial intelligence techniques—artificial neural network, K-Nearest Neighbors, Random Forest, and Support Vector Machine—were utilized to analyze the survey data. This study led to accurate predictions for the obesity classes, with success percentages of 74.96%, 74.03%, 74.03%, and 87.82%, respectively. The most effective artificial intelligence technique for this dataset was Random Forest, which had an 87.82% success rate in correctly classifying obesity [64]. In another study, Random Forests and gradient-boosted regression have recently been employed in several studies to predict postprandial glycemic responses using big data. Decision trees are essential to both gradient-boosted regression and Random Forests. Although decision trees can handle multimodal data inputs, using a single decision tree often yields subpar predictions. By creating predictions across numerous decision trees and iterations, Random Forests and gradient-boosted regression both increase accuracy [65,66]. According to another investigation by Dutta et al., the effectiveness of prediction models was evaluated using classification algorithms, such as decision trees, Random Forests (RFs), and Support Vector Machines. It was discovered that the RF algorithm predicted obesity with an acceptable accuracy level of 0.96 [67].

AI chatbots, wearables, image analysis for nutritional evaluation or obesity monitoring, and predictive modeling to evaluate the risk of obesity are other AI and ML tools utilized in obesity care [55,68,69,70]. AI models that analyze physical characteristics, eating patterns, and other health-related data, like Random Forest, XGBoost, and gradient boosting, have shown excellent accuracy in predicting obesity [71]. These AI-powered adaptive therapies have wider ramifications for public health initiatives aimed at preventing obesity than just managing individual patients. Public health professionals can create more scalable and tailored interventions that proactively target high-risk populations by utilizing real-time data and predictive analytics. Artificial intelligence (AI), like Deep Health Net, can reliably predict the risk of obesity by analyzing demographic, nutritional, and physical activity data in the early intervention strategies [72].

### 3.3. Deep Learning (DL) and Support Vector Machines (SVMs) in Obesity Prediction

Dugan et al. used machine learning techniques to predict early childhood obesity. This study used supervised and unsupervised methods such as K-means, DT, and SVM to construct a computational intelligence-based approach. Evaluation measures, including precision, recall, actual positive rate, false positive rate, and ROC area, were used to compare the approaches. After preparing and transforming the data to find missing data, unusual data, and correlation analysis, the training and classification procedures were carried out. In conclusion, the outcomes of the DT and Simple K-means approach in terms of precision (98.5%), recall (98.5%), actual positive rate (98.5%), false-positive rate (0.2%), and ROC area (99.5%) exceed those of earlier research, which had accuracy level values of 75% and 85% [73].

In another study, Abdullah et al. employed machine learning (ML) to forecast obesity in over 4000 12-year-old Malaysian children. The predictor variables, derived from questionnaires, comprised three domains: diet, physical activity, and sociodemographic. Numerous ML techniques, including BN, DT (J48), NB, ANN, and SVM, were tested in addition to numerous variable selection techniques. J48, also known as C4.5 or C5.0, is a common decision tree method for classification applications in machine learning, combined with consistent linear forward variable selection, which produces the most outstanding results. The models in this instance were not verified [74].

Nine health-related behaviors were taken from Tennessee’s 2015 Youth Risk Behavior Surveillance System (YRBSS) as model inputs. The findings indicate that DT, KNN, and ANN performed noticeably better than the logistic regression model, which had 56.02% accuracy and 54.77% specificity. In contrast to the KNN model, which produced 88.82% accuracy and 93.44% specificity, the DT model yielded 80.23% accuracy and 90.74% specificity. The ANN model performed well with 84.22% accuracy and 99.46% specificity [75].

Three machine learning algorithms were employed in Dunstan’s research to predict country-level obesity from food sales. The data was gathered from seventy-nine different nations. Their imitation confirms that they employed the five categories and that 87% of the nations they investigated could predict an obesity prevalence with an absolute error of less than 20%, 10% (regarding the entire prevalence range), and roughly 60% of the countries they considered. Their model utilizes SVM, RF, and high-gradient boosting, which are distinct machine-learning techniques for nonlinear regression used to predict obesity at the national level. Their analysis projected that the dietary categories that most closely and reliably predicted the prevalence of obesity were flour and baked goods, dairy products like cheeses, and carbonated drinks with added sugar [76].

Furthermore, Wiechman et al. used DTs in a 2017 paper that was released to shed light on the factors impacting childhood obesity among preschool-aged Hispanic children in the United States. A cohort of 2–3-year-old Hispanic children from 238 homes comprised the sample under analysis. To predict overweight, they create shallow decision trees using variables from various domains, including demographic data, parent feeding style, feeding practices, living conditions, dietary data, beverage consumption, social support, family life, the integrated behavior model, and spousal support. They discovered specific indicators of the development of obesity: the child is less likely to be overweight if the mother takes care of them or if she works while the father has a high level of education [77].

Lee’s work is an example of research that aims to understand the risk factors associated with childhood obesity. They employed DT models to predict obesity, using a South Korean longitudinal cohort of almost a million children (excluding those who are overweight). Socioeconomic status (SES, which was modeled after seeking medical attention or not), maternal factors (such as smoking, abdominal obesity, pregestational obesity, and hypertension), paternal factors (such as obesity, abdominal obesity, and hypertension), and child factors (such as preterm birth, exclusive breastfeeding, and excessive consumption of sugar-sweetened beverages) were among the 21 predictor variables of various domains that they used. The model was externally tested with a 40% test split, yielding a 93% accuracy rate [78]

Shcherbatyi’s work provides another example of successful therapeutic prediction. The efficacy of a 6-month weight-reduction therapy was predicted using a sample of 20 overweight or obese children aged 11–16 from Switzerland. They used weight, age, BMI, height, heart rate measurements, and several heart rate readings taken during a run test and a cooldown period as predictors. Many machine-learning techniques were tested, including KNN, GB, DT, and SVM. As the sample size was minimal, nested cross-validation was employed for internal validation and model training. The best model had an accuracy of 85% and was based on linear SVM. Several heart rate predictors are the most significant, according to the permutation tests they ran to determine the relative value of the predictors. These machine-learning models outperformed two domain experts’ predictions [79].

Montañez et al. identified genetic variants in the collected participant profiles using data science approaches, and these variants are then indexed as risk variants in the National Human Genome Research Institute Catalogue. Many machine-learning techniques for obesity prediction employ indexed genetic variants or single-nucleotide polymorphisms as inputs. A study on machine learning techniques for predicting obesity using available genetic profiles was conducted. To predict vulnerability to chronic hepatitis using SNP data, they utilized the SVM, decision tree, decision rule, and KNN machine learning algorithms. SVM produced the best outcome for its prediction model among all the methods. According to the outcome of their simulation, SVM produced the most significant area under the curve, measuring 90.5% [80].

A deep learning framework for predicting teenage obesity levels was proposed in this study. When compared to other models, this one performed better, obtaining high values for accuracy, F1 score, recall, and precision. Significant differences in favor of the suggested model were validated by statistical analysis. The model’s robustness was demonstrated by the fact that its efficacy was constant across gender groupings. The convergence curve demonstrated the model’s optimization, and visualizations showed the model’s superiority over the compared models in terms of producing trustworthy long-term forecasts [72].

Three AI models, such as neural networks, decision trees, and logistic regression, are among the most popular AI techniques for predicting obesity. While decision trees show nonlinear interactions and offer clear choice rules, logistic regression provides interpretable predictions and highlights important risk factors. All three strategies are highly beneficial for AI-driven obesity research, as neural networks excel at identifying subtle patterns in complex datasets and often achieve superior predictive accuracy. Among all studies in this paper, some of them are summarized in Table 1.

## 4. Discussion

Obesity has emerged as a significant public health obstacle, characterized by an excessive accumulation of body fat that poses serious health risks. In response to the global obesity epidemic, which has nearly tripled in prevalence since 1975 and affects more than 500 million individuals globally, artificial intelligence (AI)-based obesity prediction has become a critical topic of public health study [87,88]. Recent developments in AI offer intriguing approaches to improve the precision of prediction for obesity, providing creative ways to address the complexity of this health problem. Research shows that by examining a variety of datasets that include demographic, physiological, and lifestyle aspects, machine learning algorithms—such as artificial neural networks and supervised learning techniques—significantly increase the accuracy of obesity predictions [89,90]. It is crucial that ethical issues, such as algorithmic bias, data privacy, and the transparency of model outputs, are addressed to ensure equitable healthcare solutions [91].

Numerous machine learning methods have been employed in the literature to predict obesity. As supervised learning techniques, such as Random Forests and linear regression models, are widely used for classification problems, they work well. K-means clustering has performed exceptionally well in terms of accuracy, while the ANN model has shown superior performance [87]. Additionally, the ANN demonstrated the most significant accuracy in the prediction, while the RBF and SVM (perhaps more useful for clinical applications) had the most significant sensitivity [56]. In another study, ANNs and K-Nearest Neighbor (KNN) outperformed Improved Decision Trees (IDTs) and binary logistic regression in terms of accuracy [52]. Another survey indicates that the DT, KNN, and ANN performed noticeably better than the logistic regression model [75]. The best model had an accuracy of 85% and was based on linear SVM [79]. However, Lee’s study shows that the DT model yields a 93% accuracy rate in obesity prediction [78], similar to another investigation that indicates the accuracy level values of 75% and 85% are outperformed by the DT and Simple K-means technique in terms of precision (98.5%) and recall (98.5%) [73].

Studies such as Dugan et al. [73], Abdullah et al. [74], and Zheng and Ruggiero [75] demonstrate that some data, like clinical, behavioral, and school-based, can accurately predict early childhood and adolescent obesity across pediatric and adolescent populations. Other research extends AI applications to population-level datasets such as Wiechmann et al. [77] and Lee et al. [78] that used population-specific risk factors, while Dunstan et al. [76] estimated obesity prevalence using national food sales data. In addition to behavioral and demographic inputs, Montañez et al. [80] used genetic profiles to enhance obesity risk prediction, and Shcherbatyi et al. [79] used predictive models to evaluate therapeutic efficacy in obese children.Random Forests (RF) and multilayer perceptron (MLP) are also becoming increasingly popular options for obesity prediction. Creating multiple decision trees and combining their predictions, Random Forests enhance predictive accuracy, minimizing overfitting and handling large, multidimensional datasets [92]. This study demonstrates the effectiveness of decision tree algorithms in assessing obesity status and forecasting changes in the body mass index (BMI) using the ID3 algorithm, which depends on a range of input data, including lifestyle and demographic characteristics. It shows an accuracy rate of 85% and a sensitivity of 89% [92]. Random Forest was the most successful artificial intelligence method for this dataset, properly identifying obesity with an 87.82% success rate [64]. Moreover, the RF algorithm was found to have an acceptable accuracy level of 0.96 in predicting obesity [67].

The identification from the electronic health records (EHRs) included over 7000 medical visits from overweight or obese children aged 6 to 12 in several Texas sites. In order to identify EHR doctors’ activities that imply therapeutic “attention towards excess BMI”, “attention towards excess BMI comorbidities (medical risk)”, and “no attention”, they created a rule-based classification algorithm. In addition to pathology codes from EHR indexes, they utilized other types of evidence, including diagnosis codes, orders for laboratory work, prescriptions for medication, and referrals. The method was externally validated through a human assessment of 309 additional visits’ EHR data. The sensitivity of 96% was for the BMI alone, while 96.1% was for the BMI plus medical risk [93].

Gerl et al. aimed to use an advanced machine learning model to predict various obesity measures in a large population cohort based on the plasma lipidome. A unique mass spectrometric method was employed to determine the concentrations of 183 plasma lipid species in 1061 randomly selected participants from the FINRISK 2012 population cohort. A number of machine learning algorithms were developed to forecast body fat percentage (BFP), waist circumference (WC), waist–hip ratio (WHR), and body mass index (BMI). These models were then validated on 250 randomly chosen individuals from the Malmö Cardiovascular Diet and Cancer Cohort (MDC-CC). Based on a Lasso model, an analysis of the various models revealed that the lipidome predicted BFP the most accurately. Sphingomyelin molecules were the most significant positive and negative predictors, with a single double bond separating them. This suggests that an unidentified desaturase may be involved in the abnormalities of lipid metabolism associated with obesity [94,95].

An intriguing work by Nau et al., which describes a predictive model for obesogenic versus obese-protective community contexts, concludes this part. In this case, the goal is not to forecast a child’s or adolescent’s obesity but to determine whether community characteristics contribute to or mitigate childhood obesity. These authors examined 99 Pennsylvania municipalities, 50 of which had a juvenile obesity prevalence in the highest quartile and the remaining 49 in the lowest quartile. Consequently, it attempts to forecast obesogenic vs. obese protective groups using data aggregated from the community [96].

The need to create new techniques to forecast who would benefit from dietary treatments is growing as the prevalence of overweight and obesity keeps rising. AI can use computer systems that are integrated with massive data sources to solve problems. AI applications in the subject of nutrition can provide advantages, including efficient diet analysis, planning, and interpretation, high-quality nutrition advice, and a comprehensive understanding of how nutrition affects health [97].

Creating predictive models for diseases associated with obesity is another potential application of ChatGPT. Large datasets can be analyzed using ChatGPT to identify patterns and trends that may be used to predict when diseases like diabetes and cardiovascular disease are likely to manifest. Patients who are at risk of developing these illnesses may benefit from individualized preventive regimens created using this information, although further research is needed in the future [98]. In another investigation, Gerl et al. employed sophisticated machine learning in the field of metabolic markers to predict various obesity metrics based on the plasma lipidome. The study shed light on the abnormalities of lipid metabolism linked to obesity by identifying sphingomyelin molecules as key predictors and achieving substantial success in predicting body fat percentage (BFP) [94]. Interestingly, cross-validation produced a significant accuracy of 97.39%, demonstrating the model’s improved performance in estimating obesity levels with SHapley Additive exPlanations (SHAP) analysis. This approach provides medical professionals with a reliable way to gauge obesity levels [99].

Using lifestyle and individual factors from a Malaysian health survey, Wong et al. evaluated several machine learning algorithms, including logistic regression, to predict overweight/obesity status. According to this study, machine learning techniques outperformed the traditional logistic regression method. As a result, machine learning algorithms may be able to predict obesity more accurately [100]. The Obesity Prediction in Early Life (OPEL) database served as the dataset for Cheng et al. They separated the children who had clinical visits two, three, five, and eight times between the ages of 0 and 4 into male and female groups after pre-processing the data. After training an LSTM model, they achieved a lasso regression value of 0.72 and a mean absolute error of 0.98 [101].

When predicting trends in obesity prevalence based on demographic, socioeconomic, environmental, and lifestyle characteristics, their prediction model has a 91.17% accuracy rate. Furthermore, the model had a 90% accuracy rate in identifying high-risk groups. A viable method for understanding and predicting the incidence of obesity is to utilize machine learning algorithms and big data analytics [102]. There is an innovative potential for novel management and prevention tactics for childhood and adult obesity at the intersection of AI and healthcare. Personalized interventions that increase overall effectiveness, diagnostic precision, and patient accessibility can be provided by AI-powered solutions [103].

Artificial intelligence and machine learning have been used in recent studies to predict body fat and obesity across diverse demographics and data sources. Almeida et al. [81] achieved high predictive accuracy by estimating body fat percentage in school-aged children using regression and neural network models based on basic anthropometric measures. Similarly, Kupusinac et al. [83] used neural networks to predict body fat from gender, age, and BMI, demonstrating that simple inputs can yield accurate estimates.

To improve personalized weight prediction, Ramyaa et al. [82] phenotyped women based on body weight, physical activity, and dietary macronutrients using clustering and supervised machine learning. Lingren et al. [84] demonstrated the value of clinical records for automated risk detection by using an algorithm that combines structured electronic health record (EHR) data with natural language processing to identify early childhood obesity. To predict obesity at age five, Hammond et al. [85] used EHR and publicly accessible data. They achieved moderate-to-high model performance and showed promise for early-life risk assessment. Thamrin et al. [86] demonstrated the scalability of AI-based obesity prediction for public health applications by applying machine learning to population-level health survey data in adults.

**Figure 1:**
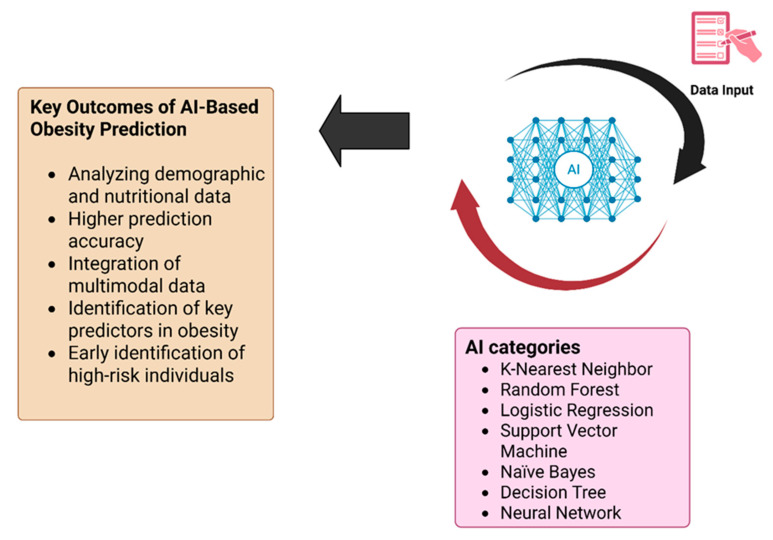
The main artificial intelligence (AI) techniques used in obesity prediction.

Although various AI and statistical techniques are used for obesity prediction, the main ones, as illustrated in Figure 1, include both deep learning and machine learning, such as logistic regression, decision tree, Support Vector Machine (SVM), and K-Nearest Neighbors (KNN) approaches.

## 5. Limitations

Studying obesity prediction with AI has some limitations, such as bias. If the training data is not representative of the general population, biases may arise. For example, if training datasets for wearables are primarily composed of data from younger individuals, the devices may produce erroneous health assessments and fail to serve older populations adequately. Furthermore, algorithmic biases may result from AI model design, which fails to consider physiologic differences among various groups, leading to discriminatory results [103]. AI models require complex algorithms to efficiently analyze massive amounts of data. Furthermore, data cleaning procedures are essential for eliminating errors or abnormalities from gathered data, which might make it more challenging to create trustworthy AI models [104]. The current review did not strictly adhere to PRISMA reporting standards. Future work would benefit from a PRISMA-based systematic approach to strengthen the completeness and transparency of the evidence synthesis.

## 6. Conclusions

Since most children with obesity become obese adults, obesity in particular is a serious global concern, and numerous studies have used statistical techniques to forecast obesity. To effectively predict adolescents at risk of becoming overweight or obese during their adolescent years, Researchers tested several well-known machine learning algorithms in this article. Additionally, the application of deep learning and machine learning (ML) approaches, such as logistic regression, decision trees, and artificial neural networks (ANNs), to the problem of childhood obesity appears to have yielded new insight into data, particularly in terms of obesity prediction. By developing AI techniques, users are more likely to adopt the Technology Acceptance Model (TAM model) by perceiving them as easy to use (perceived ease of use), helping them to make decisions, guiding and improving predictions about obesity risk (perceived usefulness), and enhancing acceptance among clinicians and patients.

The results can help analyze the relevance of methods based on computational intelligence to study different diseases or pathologies, detect them early and properly, and minimize the impact of those diseases on society.

## Figures and Tables

**Table 1:** Summary of some studies.

Lead Author, Year	Type of AI/Statistical Method	Sample Size	Input Features	Outcome Type (Classification vs. Prediction)	Finding
Ferdowsy et al., 2021 [50]	K-Nearest Neighbor, Random Forest, logistic regression, multilayer perceptron, Support Vector Machine, Naïve Bayes, adaptive boosting, decision tree, and gradient boosting classifier	~1100	Sociodemographic, lifestyle variables	Classification	Logistic regression was the best-performing algorithm with an accuracy of 97.09%, although regression modeling may not be able to effectively untangle interdependent and nonlinear interactions.
Ergün et al., 2009 [59]	Logistic regression and neural network	~82	Body mass index (BMI), divergent arteries	Classification	Both of these systems were effective classifiers for detecting obesity, although the classification rate of neural networks is about 90%, and the classification rate of logistic regression for obesity is nearly 87%.
Heydari ST et al., 2012 [60]	Logistic regression and neural network	~414	Socioeconomic status, anthropometric measures	Classification	Although logistic regression and neural networks were both effective classifiers for detecting obesity, there was no discernible difference between them in classification, as sensitivity for both of them is 80.2% (LR) vs. 79.7% (ANN), and area under ROC is 0.888 (LR) vs. 0.884 (ANN).
Zhang et al., 2009 [56]	Decision tree, artificial neural networks, Support Vector Machines, radial basis function, Naive Bayes.	~16,523	Early growth records (data at birth, 6 weeks, 8 months, and 2 years)	Classification	The RBF and SVM (perhaps more beneficial for clinical applications) had the most significant sensitivity in the prediction at eight months, whereas the ANN showed the most considerable accuracy. Logistic regression had the highest specificity compared to machine learning models, but its sensitivity and accuracy were significantly lower.
Lazarou et al., 2012 [57]	Logistic regression models with PCA	~1140	Dietary data, consumption frequencies of specific food groups	Classification	As predictor variables, they employed questionnaires about the frequency of consumption of several food types; only one of the PCs of the girl’s model was significant.
Pochini et al., 2014 [58]	Logistic regression and decision tree		Lifestyle risk factors (physical activity, diet)	Classification	The logistic regression model identified significant predictors.
Duran et al., 2019 [61]	Artificial neural networks	~1999	Age, height, weight, waist circumference (WC)	Classification	Artificial neural networks (ANN) outperform simple models—particularly the z-WC model—in terms of accuracy, sensibility, and specificity, and regarding girls, ANN outperforms both the z-BMI and z-WC models.
Ríos-Julián et al., 2017 [52]	Decision tree, Random Tree, Random Forests, Naïve Bayes, and Bayes Net		Anthropometric, simple risk questionnaire items	Classification	ANNs and K-Nearest Neighbor (KNN) performed more accurately than binary logistic regression and Improved Decision Trees (IDTs). ANNs, logistic model trees, and basic logistic regression performed better in accuracy than J48.
Koklu et al., 2024 [64]	Neural network, K-Nearest Neighbors, Random Forest, and Support Vector Machine	~1610	Lifestyle variables such as social activity, physical activity	Classification	The most effective artificial intelligence technique for this dataset was Random Forest, which had an 87.82% success rate in correctly classifying obesity. However, accuracy for ANN, KNN, and SVM was 74.96%, 74.03%, and 74.03%, respectively.
Dutta et al., 2024 [67]	Decision trees, Random Forests, and Support Vector Machines.	~2111	BMI, family history of overweight, age, weight, and eating behavior variables	Classification, prediction	It was discovered that the RF algorithm predicted obesity with the highest accuracy level of 0.96. Other algorithms’ accuracy was ~95% for the decision tree and ~88% for the SVM.
Dugan et al., 2015 [73]	Random Tree, Random Forest, J48, ID3, Naïve Bayes, and Bayes	~7519	CHICA (Child Health Improvement through Computer Automation) survey questions	Classification	The outcomes of the DT and Simple K-means approach in terms of precision (98.5%), recall (98.5%), actual positive rate (98.5%), false-positive rate (0.2%), and ROC area (99.5%) exceed those of earlier research, which had accuracy level values of 85%.
Zheng et al., 2017 [75]	Decision tree, K-Nearest Neighbors, and artificial neural networks, logistic regression	~5127	Health-behavior variables/risk + protective factors	Predicting	The findings indicate that DT, KNN, and ANN with an accuracy of 80.23%, 88.82%, and 84.22%, respectively. Decision tree (IDT) accuracy: ~performed noticeably better than the logistic regression model, which had 56.02% accuracy and 54.77% specificity.
Wiechman et al., 2017 [77]	Decision tree		Demographic data, parent feeding style, living conditions, dietary, social support, family life, behavior model, and spousal support	Prediction	To predict overweight, they create a shallow decision tree.
Lee et al., 2019 [78]	Decision tree	~1,001,775	Parental factors, child-related factors	Classification	The model was externally tested with a 40% test split, yielding a 93% accuracy rate.
Shcherbatyi et al., 2018 [79]	K-Nearest Neighbors, decision tree, and Support Vector Machine		Age of the child, heart rate, and other current parameters collected before therapy	Classification	The best model had an accuracy of 85% and was based on linear SVM.
Montañez et al., 2017 [80]	Support Vector Machine, decision tree, K-Nearest Neighbors, Random Forest, and other models		Genetic data	Prediction	SVM produced the best outcome for its prediction model among all the methods. According to the outcome of their simulation, SVM produced the most significant area under the curve, measuring 90.5%.
Almeida et al., 2016 [81]	Regression models and neural networks	~3084	Age, sex, anthropometric measurements, BMI, weight, height, waist and hip circumference, waist-to-height ratio, waist–hip ratio, skinfolds	Classification, prediction	Anthropometric parameters, excluding skinfold thickness, can be used to predict body fat percentage with a reasonable degree of accuracy (≥91.3%).
Ramyaa et al., 2019 [82]	Support Vector Machine, and K-Nearest Neighbors	~48,508	Dietary intake, physical activity, demographics/health	Prediction, clustering	SVM regression was the most appropriate predictive technique, closely followed by KNN and neural network methods. Clustering yielded comparatively better fit statistics, even if the total data model demonstrated a decent fit and predictive capabilities.
Kupusinac et al., 2014 [83]	Artificial neural network	~2755	Gender, age, BMI	Prediction	An artificial neural network (ANN)-based program solution for BF% prediction is presented in this paper. The ANN’s output is BF%, while its inputs are GEN, AGE, and BMI. This paper shows an 80.43% prediction accuracy.
Lingren et al., 2016 [84]	Machine learning	~650	ICD-9 diagnosis codes, RxNorm medication codes, demographic data, height, and weight	Classification	Machine learning methods were built through cross-site training and testing with high precision. Overall, the rule-based approach scored the highest at 0.895 in Cincinnati Children’s Hospital and Medical Center (CCHMC) and 0.770 in Boston Children’s Hospital (BCH). ICD-9 codes, RxNorm codes, and Unified Medical Language System (UMLS) concept unique identifiers (CUIs) were used in the optimal feature set for machine learning.
Hammond et al., 2019 [85]	Machine learning	~3449	EHR-derived features, maternal data, neighborhood/socioeconomic data	Classification	The ability of academics and clinicians to influence future policy, intervention design, and clinical decision-making could be enhanced by machine learning techniques for forecasting pediatric obesity using electronic health record (EHR) data that has an area under the Receiver Operator Characteristic Curve (AUC).
Thamrin, 2021 [86]	Logistic regression, Classification and Regression Trees (CART), Naïve Bayes	~618,898	Risk factors available in the previous study (RISKESDAS survey)	Classification	Using publicly available health data, machine learning (ML) techniques like logistic regression, Classification and Regression Trees (CART), and Naïve Bayes are used to detect the presence of obesity, predict obesity using a novel approach with advanced ML techniques in an effort to surpass conventional prediction models, and compare the effectiveness of three different approaches. Additionally, they use the Synthetic Minority Oversampling Technique (SMOTE) to predict obesity status based on risk factors found in the dataset in order to resolve data imbalance. The method with the best results is logistic regression.

## Data Availability

No new data were created or analyzed in this study.
